# Development of a detection chip for major pathogenic drug-resistant genes and drug targets in bovine respiratory system diseases

**DOI:** 10.1515/biol-2022-0778

**Published:** 2024-03-26

**Authors:** Jie Qi, Penghui Li, Yasong Yan, Gongmei Li, Lingcong Kong

**Affiliations:** College of Veterinary Medicine, Jilin Agricultural University, Changchun, China

**Keywords:** bovine respiratory disease syndrome, TaqMan MGB fluorescence quantification, QRDR, detection chip

## Abstract

Bovine respiratory disease (BRD) is a significant veterinary challenge, often exacerbated by pathogen resistance, hindering effective treatment. Traditional testing methods for primary pathogens – *Mycoplasma bovis*, *Pasteurella multocida*, and *Mannheimia haemolytica* – are notably time-consuming and lack the rapidity required for effective clinical decision-making. This study introduces a TaqMan MGB probe detection chip, utilizing fluorescent quantitative PCR, targeting key BRD pathogens and associated drug-resistant genes and sites. We developed 94 specific probes and primers, embedded into a detection chip, demonstrating notable specificity, repeatability, and sensitivity, reducing testing time to under 1 h. Additionally, we formulated probes to detect mutations in the quinolone resistance-determining region, associated with fluoroquinolone resistance in BRD pathogens. The chip exhibited robust sensitivity and specificity, enabling rapid detection of drug-resistant mutations in clinical samples. This methodology significantly expedites the diagnostic process for BRD and sensitive drug screening, presenting a practical advancement in the field.

## 1 Introduction

Bovine respiratory disease (BRD) has consistently posed a substantial challenge in the realm of beef cattle farming, inflicting notable economic repercussions globally, approximated at $300 million per annum. Solely in the UK, diseases of the respiratory system impact 1.9 million cattle, culminating in the demise of 157,000 individuals [1–3]. Research conducted by Gagea et al. [4] reveals that a substantial majority, over 50%, of respiratory diseases are attributed to mixed infections, predominantly involving *Mannheimia haemolytica* and *Mycoplasma* species. An epidemiological study in Ontario, Canada, executed by Hotchkiss et al. [5], discovered that respiratory tract diseases were the cause of death in 76% of cattle cases. Further, upon conducting pathogen detection in 54 pneumonia-afflicted cattle, *Mycoplasma bovis* was present in 53 cases, with numerous instances (19/53) also exhibiting concurrent infection with *Histophilus somni*, and others with *Trueperella pyogenes* (14/53), *M. haemolytica* (11/53), and *Pasteurella multocida* (9/53). A study by Pardon et al. [6] on Scottish cattle farms identified *M. haemolytica* as the causative agent in 17% of respiratory system diseases. Additionally, Xiao et al. [7] isolated pathogens from cattle experiencing respiratory diseases in Belgium, reporting isolation rates of 70.5% for Mycoplasma species, 21.5% for *H. somni*, and 26.0% for *M. haemolytica*.

Nonetheless, the principal pathogens correlated with BRD in China exhibit slight variations compared to those in international contexts. Predominantly, Mycoplasma species and *M. haemolytica* emerge as the chief pathogens in China, with a reduced incidence of mixed infections involving alternative pathogens. A study by Caruso and Ross [8] involved the collection of clinical samples from 35 cattle, diagnosed with primary *Mycoplasma pneumonia* across the nation. The isolation and identification of pathogens revealed a dominance of *Mycoplasma* species infection, with mixed infections primarily comprising *Mycoplasma* species and *M. haemolytica* Type A.

In addition, pertinent studies indicate that porcine respiratory mycoplasmas, sharing the same genus as bovine respiratory mycoplasmas, possess the capability to inflict damage on the respiratory tract. These mycoplasmas engage in interactions with bacteria such as *Bordetella*, *Streptococcus*, and *Pasteurella*, resulting in exacerbated lesions and an extended disease duration. Marois et al. [9] documented that simultaneous infections of *M. haemolytica* and *P. multocida* further diminish macrophage functionality and hypothesized that mycoplasmas might inhibit macrophage capability, thereby heightening the host’s susceptibility to secondary bacterial infections. A study conducted by Wang et al. [10] utilizing specific pathogen-free pigs revealed that an isolated infection with *Actinobacillus pleuropneumoniae* (APP) serotype 9 typically manifested subclinical symptoms. However, when pigs were pre-infected with *M. haemolytica* 4 weeks prior to APP infection, clinical symptoms of APP were evident, signifying that *M. haemolytica* enhances the pathogenicity of APP.

According to pertinent research, the confluence of *Mycoplasma* species and *M. haemolytica* within the lungs precipitates more severe lesions. Epithelial cells within respiratory organs serve as the initial physical barrier against external bacteria. Upon infection, bacteria must first engage with, invade, and dismantle the epithelial cells to penetrate the host’s tissues, subsequently inducing pneumonia and related diseases. Once adhered to host cells, mycoplasmas can disrupt the functionality of cell membrane surface receptors. For instance, porcine respiratory mycoplasmas can impair K^+^ channels in ciliated bronchial epithelial cells, resulting in cilia stasis. Moreover, mycoplasmas generate various toxic metabolites, such as cytolysins and superoxide radicals. The membrane-bound phospholipase in mycoplasmas catalyzes the hydrolysis of host cell phospholipids, disrupting the host cell’s signal transduction pathways. Resultant hemolysins can also compromise cell membrane integrity. Additionally, research indicates that mycoplasma infection typically triggers the production of prostaglandin E2, which inhibits neutrophil activity and may subsequently induce secondary bacterial infections [11–13].

Consequently, there is an imperative need to develop mutation detection methodologies for the primary pathogens and drug resistance genes of BRD, as well as routinely utilized drug resistance targets. This study pioneered a gene chip technology that is swift, efficient, continuous, and precise [14]. In recent years, gene chip technology has been recurrently employed for the identification and categorization of pathogenic microorganisms. Through the screening of pivotal genes, the microbial genome can be directly analyzed, facilitating the identification and classification of bacterial species. Anthony et al. [15] utilized gene chip technology to expedite the diagnosis and identification of bacterial cultures within 4 h, achieving an accuracy rate of up to 77.8%. El-Sayed and Kamel [16] employed the same gene chip to detect genes representing specific bacterial species, successfully accomplishing bacterial identification and classification. In China, Zhai and Guo [17] developed gene chips for over 20 bacteria, including *Escherichia coli* and *Salmonella*, successfully identifying clinically prevalent infectious bacteria. Aslam et al. [18] utilized oligonucleotide microarrays to detect mutations in the gyrA gene of three pathogenic bacteria.

Numerous diseases originate from genetic factors, and conventional molecular biology techniques often fall short in elucidating the interactions and sequential information among multiple genes during biological processes. Gene chip technology emerges as a remedy to these limitations, offering early and precise diagnosis and treatment for certain diseases, particularly in identifying highly pathogenic genes. Heller et al. [19] explored the tissue genes of enteropathic rheumatoid arthritis utilizing gene chip technology, pinpointing IL-3, Gro-A, and other pertinent genes through the analysis of differentially expressed genes. Wang et al. [20] employed gene chip technology to sift through genes associated with drug resistance in tumor cells. Furthermore, Yao et al. [21] leveraged gene chip technology to scrutinize gene expression in breast cancer, unveiling two novel carcinogenic genes: H2AFJ and EPS8.

In light of the primary pathogens of bovine respiratory system diseases, prevalent drug resistance genes, and common mutation sites in fluoroquinolone drugs, this study meticulously crafted a gene chip, embodying a pivotal advancement in the realm of veterinary medicine, especially concerning BRD. This innovation is not merely a diagnostic tool but a comprehensive system that facilitates systematic, swift, and high-throughput detection, thereby significantly enhancing the identification of major pathogens and the screening of sensitive drugs for BRD. The precision and accuracy in detection, underscored by the chip’s ability to target specific genes, ensure that the identification is not only rapid but also highly accurate, minimizing the risk of false positives and negatives, which is paramount for effective disease management. Furthermore, the high-throughput capabilities of the gene chip enable it to process and analyze multiple samples simultaneously, which is vital for managing BRD on a larger scale, such as in farming industries, where timely detection and management can prevent widespread outbreaks and minimize economic losses. The rapid detection offered by the gene chip is a pivotal advancement, providing timely intervention that can save lives and resources, while its enhanced drug screening functionality ensures that the management of BRD is not only reactive but also proactive, thereby providing a comprehensive solution to managing the disease. Moreover, the gene chip has practical applicability and can be seamlessly integrated into real-world scenarios, such as veterinary clinics and farms, ensuring that the benefits of the technology extend to where it is most needed. The development of the gene chip also paves the way for further research and development, providing a foundational technology that can be adapted and evolved to manage other diseases and conditions, thereby contributing not only to the immediate field but also providing a springboard for future advancements in veterinary medicine and beyond.

## 2 Materials and methods

### 2.1 Strains and clinical samples

Isolates A, B, D, E, and F types of *P. multocida* (Pm), *M. haemolytica* (Mh), *M. bovis*, *Streptococcus* spp., APP, and *Haemophilus parasuis* from cows were identified and preserved in our laboratory. From a cattle farm in Jilin Province, China, a total of 97 nasal swabs were gathered from BRD suspected clinical cases.


**Ethical approval:** The research related to animal use has been complied with all the relevant national regulations and institutional policies for the care and use of animals.

### 2.2 Selection of target genes

Currently, *P. multocida*, *M. bovis*, and *M. haemolytica* are recognized as the predominant pathogens of BRDs in China. In recent years, the drug resistance spectrum of these pathogens has progressively expanded. Consequently, this experiment was undertaken, targeting seven drug resistance genes and a range of pathogens commonly associated with BRD for the high-throughput detection chip. These pathogens include bovine podococcal A, B, D, E, and F type Pm, bovine *M. bovis*, bovine Mh, porcine *M. pneumoniae*, *Streptococcus*, *Haemophilus parvum*, and bronchial septic bacillus.

### 2.3 Primer and probe design

Conserved regions across 81 antibiotic resistance genes and 13 pathogenic bacterial species were identified by selecting their full sequences from the NCBI database. Multiple alignments were executed using BLAST, and specific primers along with TaqMan-MGB probes were crafted for the conserved regions, adhering to the principles of primer and probe design via Premier 5.0 software. The probes were marked with a fluorescent group FAM at the 5ʹ end and a quencher group MGB at the 3ʹ end. The design principles for the probes are as follows:

1. The first nucleotide at the 5ʹ end of the probe cannot be G.
2. The probe should be as short as possible, but not less than 13 nucleotides.
3. The Tm value of the probe should be designed between 65 and 67°C.
4. The same nucleotide should not repeat more than four times, especially guanine (G).
5. The probe should contain more cytosine (C) than guanine (G).

After designing the probes, specific primers for the target genes were designed according to the following principles:

1. The primers should be as close as possible to the probes, but they should not overlap.
2. The GC content should be maintained between 30 and 80%.
3. Among the last five nucleotides at the 3ʹ end, the sum of G and C should not exceed 2.
4. The amplicon size should be between 50 and 150 bp.

The designed specific primers and MGB probes were synthesized and embedded into a solid-phase template by Thermo Fisher Scientific (China) Co., Ltd.

### 2.4 Vector construction and transformation

The antibiotic resistance gene sequences were ligated to pMD18-T vector and transformed into competent *E. coli* DH5α cells by heat shock to construct cloning vectors. The pMD18-T vector connection system is shown in Table 1.

**Table 1:** pMD18-T carrier connection system

Component	Amount (μL)
DNA	5
Solution I buffer	4.5
pMD 18-T	0.5
Total	10

### 2.5 Extraction of recombinant plasmids

About 1.5–5 mL of overnight culture was collected in an EP tube, centrifuged at 8,000×*g* for 2 min, and the supernatant was completely removed. Plasmid extraction was performed according to the instructions of the plasmid extraction kit from Beijing Saibaisheng Gene Technology Co., Ltd. The obtained plasmid DNA was stored at −20°C for future use.

### 2.6 Preparation of standard solutions

The concentration of the extracted plasmid DNA was determined using a UV spectrophotometer, and the purity of the plasmid DNA was assessed by the OD260/OD280 ratio. If the ratio was between 1.6 and 1.8, the purity of the extracted plasmid was considered satisfactory for the construction of a standard curve. The copy number of the plasmid was calculated using the formula: Plasmid copy number (copies/μL) = OD value (ng/μL) × (6.02 × 1023) × 10^–9^/(plasmid DNA base pairs × 660). The plasmid was diluted ten-fold with Elution Buffer and stored at −80°C.

### 2.7 Optimization of TaqMan MGB fluorescent quantitative PCR reaction conditions

The optimization of the TaqMan MGB fluorescent quantitative PCR system aimed to reduce non-specific hybridization during the experiment. In this study, 91 constructed plasmid standards were used as templates to optimize the reaction conditions. The annealing temperature was gradually increased from 55 to 60°C in steps of 1°C, and PCR amplification was performed. The obtained Ct values and curve shapes were analyzed to select the optimal reaction system and conditions based on the recommended conditions of Thermo Fisher Scientific (China) Co., Ltd.

### 2.8 Construction of standard curves

Using the optimized PCR system and conditions, plasmid standards with concentrations of 1 × 10^10^ copies/μL, 1 × 10^8^ copies/μL, 1 × 10^6^ copies/μL, 1 × 10^4^ copies/μL, and 1 × 10^2^ copies/μL were used as templates for fluorescent quantitative PCR. Each group was tested in triplicate to construct the standard curves, and data analysis was performed based on the curve.

### 2.9 Specificity of detection for antibiotic resistance genes and pathogens

Plasmid standards of *M. b*ovis, *Streptococcus* spp., and APP were successfully constructed and adjusted to a concentration of approximately 1 × 10^8^ copies/μL. Plasmid standards of *M. bovis* and *P. multocida* were used as positive controls to assess the specificity of the TaqMan MGB fluorescent quantitative PCR detection chip.

### 2.10 Sensitivity of detection for antibiotic resistance genes and pathogens

The constructed plasmid standards were diluted ten-fold to a concentration ranging from 1.0 × 10^1^ to 1.0 × 10^10^ copies/μL. TaqMan MGB fluorescent quantitative PCR reactions were performed to determine the lowest detectable concentration of the plasmid standards by the chip.

### 2.11 Reproducibility of detection for antibiotic resistance genes and pathogens

Plasmid standards of *P. multocida* A type and *M. haemolytica* with concentrations ranging from 1.0 × 10^4^ to 1.0 × 10^8^ copies/μL were selected as templates for inter-batch and intra-batch reproducibility tests. The average Ct (MN), standard deviation (SD), and coefficient of variation (CV) were calculated based on the obtained Ct values. The calculation formula for the CV was CV% = SD/MN × 100%.

### 2.12 Clinical sample detection

To evaluate the clinical applicability of the high-throughput detection chip established in this study, 97 bovine nasal swab samples collected from a cattle farm in Jilin Province were tested. The collected samples were evenly spread on LB solid medium containing ampicillin in a biosafety cabinet. The plates were incubated overnight at 37°C, and suspected positive colonies were picked and transferred to LB liquid medium containing ampicillin. The liquid cultures were shaken at 37°C for 6–12 h. Then, 1 mL of bacterial suspension was used as the template for clinical detection.

## 3 Results

### 3.1 Synthesis of primers and probes

In this study, based on the gene sequences provided in the NCBI database, we designed and synthesized 81 specific primers and probes for seven classes of drug-resistant genes and 13 probes and primers for seven common pathogen species associated with bovine respiratory system diseases, following the principles of probe and primer design (Table 2).

**Table 2:** Primers and probe sequences for TaqMan MGB fluorescence quantitative PCR amplification

Assay name	Primer sequence		Probe sequence
	F	R	
*aacA/aphD*	AGAGCCTTGGGAAGATGAAGTTTTT	CTATCTCATCAGTTTTTGGATAATGATAATCAGTATATAACTC	CCATATCCAATAGGAACATTG
*aph*(*2ʹ*)*-Id-01*	GACAGAACAATCAATCTCTATGGAATGT	GAGCAGTATCATAAGTTGAGTGAAAAGG	ACGTCGCTTCATCATATG G
*aph*(*2ʹ*)*-Id-02*	CCTCTTCATACCAATCCATATAACCATATTCC	AAGGATATACCGACAGTTTTGGAAAA	TCGAACGACCAGTATTTT
*aac*(*6ʹ*)*-Iy*	GGAGAACAAAAATACCTTCAAGGAAAGC	CCGCCACGATTATGTCAATGG	ACGGGCGAACTGTCAC
*aac*(*6ʹ*)*I1*	CGGATTAAGGCCGATGTACGAT	GCCTTGATATTCAGTTTTTATAACCATGGG	AAGACCTGGGAACTTC
*aacC1*	GCAAGTTCCCGAGGTAATCG	GGTCGTGAGTTCGGAGACGTA	CCACCTACTCCCAACATC
*aadA-01*	CTCGAAGATACCTGCAAGAATGTCA	TTATCCAGCTAAGCGCGAACT	CCATTCTCCAAATTGC
*aadA1*	GCTCGAAGATACCTGCAAGAATGT	GCGCGAACTGCAATTTGGA	CATTGCGCTGCCATTC
*aadA-1-01*	CTTTCACAAAGATGTTGCTGTCTCC	GCCCGAAGAGGAACTTGTCT	TTCCCACGGCGACCTG
*aadA2-01*	CGGCTCCGCAGTGGAT	GCCACAGTAACCAACAAATCA	ATATCGCTGTATGGCTTCAG
*aadA5-01*	CTGCGGATGGGCCTAGAAG	TCACGATCTTGCGATTTTGCT	AAGGCGAGGCAACACA
*aadA5-02*	AGGCAAACGCTCCGATACC	ACTGGTCTCATTGCTCCTAAGGA	CATGCGGCAGCAACG
*aadA9-01*	CGCGGCAAGCCTATCTTG	CCAATGAACGCCGAAGTCTCA	CTGCACGCAAAGCAA
*aadD*	AGCGCTCGTCGTATAACAGATG	CCTTGACTGTACAGGTAGCAATGG	ATGCAGACCAATCAAC
*aadE*	GGAACTATGTCCCTTTTAATTCTACAATCT	TGCCCTTGGAAGAGTTAGATAATTACCT	AAAGGGCGATAAATTAAT
*aph*	CCAAGCTGTTTCCACTGTTTTTCTG	CAGCAAGTGGATCATGTTAAAATAATTGTGT	ATGCGCCCAATGGTT
*aph6ia*	CCCATCCCATGTGTAAGGAAATT	CACCGCTTCTGCTGTACGA	TCGTCGGACCACATCCA
*aphA1*(*aka kanR*)	ACCATGAGTGACGACTGAATCC	TGAACAAGTCTGGAAAGAAATGCA	AAGCTTTTGCCATTCTC
*aphA3-01*	CTTTCACAAAGATGTTGCTGTCTCC	GCCCGAAGAGGAACTTGTCT	TTCCCACGGCGACCTG
*aphA3-02*	TCCCACCAGCTTATATACCTTAGCA	CGGAATTGAAAAAACTGATCGAAAAAT	ACCGCTGCGTAAAAG
*sul2*	CCGCAATGTGATCCATGATGTC	CCAAACTCGTCGTTATGCATTCG	CCTCGCGCCGATCTG
*strA*	GCTTAAAATGAGAGATAGACCGGAACA	GTAAGTCCGAGAACATGCTTTCC	CCGGTGCAAGACCAT
*strB*	CGGTCGTGAGAACAATCTGATGT	GGCAACGATGTGAGAGAGCAT	TCGCTCCCCGGCATAT
*dfrA1*	GCCCTGATATTCCATGGAGTGC	CGTCCAACCAACAGCCATTG	CAGGAGCTGTTCACCTTT
*dfrA12*	GCGACAGCGTTGAAACAACTAC	CGAACCGTCACACATTGGTAATCT	CACGCCAAGCTAACTAC
*folA*	CCCAGTCATCCGGTTCATAATCC	GCAGAAGCTTTATCTGACGCATATT	ATCGCCTTCGACTTCC
*tet*(*34*)	CTTAGCGCAAACAGCAATCAGTT	GGTGATACAGCGCGTAAACTAC	TCGCTTTCGGGTACATTT
*tet*(*35*)	CAACCCACACTGGCTACCA	GTACCTGTAGAGAACGCCATTAGG	CCAGACAGCAAGAACA
*tet*(*36*)*-01*	TCAGCAGAGGTCAGTTCCTACA	TGGTAGGTCGATAACCCGAAAATC	ACGCCCAAGCCTTGTG
*tet*(*36*)*-02*	CAGGAAAGACCTCCATTACAGAGAA	TTTGTCCACACTTCCACGTACTATG	CTCCACTCGCAAATAG
*tet*(*37*)	GAGAACGTTGAAAAGGTGGTGAAC	ACCAAGCCTGGATCAGTCTC	ATGATCGTATGTCGAAATAT
*tet*(*38*)	GCCTGGGAAATTTAATGCTTTAAAATCGA	TGGCGGTATCTGTAGGTATTGC	TAGAGCCGCAGCAATC
*tetA-02*	CACGTTGTTATAGAAGCCGCA	CAGCCTGACCTCGATCGT	TCCTCTTCACGGCGATCTA
*tetB-01*	GCCCCAGTAGCTCCTGTGA	GTGCGCTTTGGATGCTGTATT	CCCTGAAAGCAAACGGCCTA
*tetB-02*	TGAAAGCAAACGGCCTAAATACAG	CGCATCGCTGGATTACTTATTGC	TCCAAAGCGCACTTGAA
*tetE*	TTGGCGCTGTATGCAATGATG	CGACGACCTATGCGATCTGA	TTTGCCCCTCTTCTCGGC
*tetG-01*	TGCCCGCCCCATAACAG	GAAGGTTCTCGCGCACG	CCATGTAGCCGAACCAG
*tetG-02*	CAATGGTTGAGGCTGCTACAG	CGGTCTTATGGGTGCTCTATATCG	CCGTGACGCCGGACAC
*tetH*	GCGCATTATCATCGACAGATTTTGA	GCTTAGCGGCAGGAGGTAT	ATGCGGGTTGCCCC
*tetL-02*	TCCCATGGCTACTATCGATCCAATA	GTAGTTGCGCGCTATATTCCAAAG	ATGCTTTACCCCTATTTTC
*tetK*	CAGCAGTCATTGGAAAATTATCTGATT	CAAAATAAAAAAGTGATTGTGACCAATAAAAGCA	CCAAGACAGCTCAAACTA
*tetL-01*	CGCAACGACAACCATCACA	GCCCGATTTATTCAAGGAATTGGT	CCGCATTCCCAGCTCT
*tetM-01*	CGCCATCTTTTGCAGAAATCAGTAG	CAGGACATATGGATTTCTTAGCAGAAGT	TTGCCCCATCTAAAACT
*tetM-02*	CCGTCCTCGTTGTACCTTTGTC	AGAAAGCTTATTATATAACAGTGGAGCGATT	ACGCTTCCTAATTCTG
*tetO-01*	CTCAAGGATGGCACAAATGACTTC	TGTGGATACTACAACGCATGAGATT	CATCTGCACATTCCC
*tetO-02*	TGTCCTTGTTGTGCCTTCATCT	GAAAGTTTATTGTATACCAGTGGTGCAA	ACGCTCCCTAGTTCTGC
*tetPA*	TGCTACAAGTACGAAAACAAAACTAGAAA	AGTTGCAGATGTGTATAGTCGTAAAC	CAGGAGTGGGATTTAT
*tetPB-02*	TGATACACCTGGACACGCT	CGTCCAAAACGCGGAATGATC	CTCCACTTCAGCGATAAAA
*tetPB-03*	GGCGACAGTAGGCTTAGAAATAGAA	GACCCTACTGAAACATTAGAAATATACCT	ACCTTCGCCTCTCCC
*tetPB-04*	GGTGCAAATACTGAAAAAGTTGTAAAGCA	TTGTTCCTTCGTTTTGGACAGAAT	CAAATGAAGCATTCCCC
*tetPB-05*	TGAAGTGGAGCGATCATTCCG	CCCTCAACGGCAGAAATAACTAAA	ATCGCACCGTCCAAAAC
*tetQ*	GGCTTAGGCGTTTTTATGGTCAAG	TGCGGATATTATCAGAATAATCGCCTTT	CCATGCGGGTATCAAA
*tetR-01*	ATGAGTTCGGCCAGAATTTCCT	GGTTGTGCGCGAAATGATTT	TCGGCGACCACGCGAC
*tetR-02*	CTTTTCGCCAATCCATCGACAA	CGGACGCAGCGTTCGA	TCACCGCGAGTCCCT
*tetR-03*	CGCGATGGAGCAAAAGTACATTTAG	GCTAATTGATTTTCGAGAGTTTCATACTGT	ACACGGCCTACAGAAAA
*tetS*	AGGACAAACTTTCTGACGACATCAT	TCTCCCATTGTTCTGGTTCAGTATAATCTA	AAGCAGACTGTGAATCTA
*tetT*	CCATATAGAGGTTCCACCAAATCCT	GACCCTATTGGTAGTGGTTCTATTGA	CAGTCCAATAGATGCCC
*tetU-01*	GTGGCAAAGCAACGGATTGG	TGCGGGCTTGCAAAACTATCT	AAGCTTTCCTGAACCATCG
*tetW-01*	AGCTTATCCCGAACAGACTGAAC	CATTCCCACCGTTATCTTTATCAACAAG	ACGCTCTGCAAATCA
*tetU-02*	GGGTTAAGTGTGCAAGGTACGA	CAGTTTTCCGACAATTGTAATTCGATCA	CACCCCCCTAAAATT
*tetV*	CTCACGACCATGATGCTGATGT	CGACGATGTATATCCCACGATCAC	TCGGCTCGATTCCCCT
*tetX*	CATAGCTGAAAAAATCCAGGACAGTT	CACGGAAGTTGAAGAAACAGGTACT	CTGGTTGATGAATATCG
*TETX4*	CAGAAATGACTTAAGGGCTATCTTGTTGA	ACTTCTTCTTACCAGGTTCAAGCAT	ACGACACGGTTATTTG
*TETX3*	GGTGTAAATATTGTTGATGAAAAGGGCAA	TTCTGTTTATTTCAGGATTGTCAAAACGATTT	TCGGGCCTTACATTTT
*kmt1*	ACCGGCAAATAACAATAAGCTGAGT	AGCCAATCTGCTTCCTTGACA	ACGGCGCAACTGATTG
*PASTEURELLA-2*	TGCCAAAACTTCTTAACATTACACCATCT	TGTTGATGGACGTTGTAAAGACTGA	ACGGAGTACCAATTTT
*PASTEURELLA-3*	TCATAGAATGATTAAATACTATGGTAAAAATAGGATAAATAACTT	CATCTACCCACTCAACCATATCAGAA	CAATGCGTGAAGATTC
*PASTEURELLA-4*	TCCCCAACTCAACTTCATGAAATTGT	GCGCTAAGCGAGCATGTG	CCCAACGATCATTTTC
*PASTEURELLA-5*	GTACAGCAAAGTATGATTTTGTCTCGAT	TCTTCTAATAGTTCTGTAAGATAAGAATGAACCCA	ATGGCACCACAACAAT
*MBoppd*	GGGCGAAGATGTAGAATTTGGTTAC	TCCGCCGTCAATTACTCTGAAAA	CCTTTGGCAAATAATCT
*MB16s*	CTAACAAAAACGCTTTTAATAATTTTCTTTCGGAA	TCTATGTCGTAAGTATTTAATCTTGCATAACGG	ACGAGATCAAAATTTG
*MCATTCE*	CGGTGAAGCCTTTGACAAAACAG	TCGGCTAATTTTGACATCGCTACA	TCGGTTTGGATTACCC
*MHY-GENE*	CCTTTAAGACTGGGATAACTATTGGAAACA	GAAGCTGTGAAGCTCCTTTCTATTAC	CATCATGCGATAAATAAC
*HPARA*	TGGCTTAGATGATTGGGACAAATGT	AGCCCCTGGCACTGC	AACGCAGGATAGCTTG
*STREPTOCOCCUS*	CGAAGAAGAACACCAACGTTGTC	CTGGTGTTGAAATGTTCCGTAAACA	CCCTGCAAGACCTTC
*ABRAC*	CGCACATTTCCGAACTTCACTTTT	GATTTCCTTTGTTGCCTGGATTACG	TCCGTCGCAAACCT
*ERMB*	ACACTCAAGTCTCGATTCAGCAATT	GGCGGGTAAGTTTTATTAAGACACTGT	CCAGCGGAATGCTTT
*CATB*	GGTCAGACGTTCCATTGCATCA	GGTGGCATTGATCTGATCGAACA	CCTTGCGCCATTAAC
*QNRA*	GCGCGATGCCAGTTTCAAG	GTTGGCACCGCTGAAGTTG	CTGCCGTCTGTCTTTG
*ACRA*	GCGAAAGCTGCCGTTGAA	CGGAGAGGTAACTTTGGTGTAAGC	ACTGCGCGAATCAA
*ACRB*	CGGCGGCGGTTCTG	CGTTTAAATGCCCACTTGACTTTTG	TTGCTTGGCTTCTTCC
*FLOR*	TGGGAGCAGCTTGGTCTTC	CCACTGCTTGAAGTAGACGGAAAG	CTGCACCGGCCTTTGT
*FEXA*	TCTGTTGTAGCTTTGGTGGGATTT	GTTATTGAACAGGACAGGTGGTACA	ACCGCAGAAAATCCAT
*OPTRA*	CGTAGTATGGGTTTTACTGAAGCAGAT	TCATCAAGTAATAGAATGTCTGGCTTTGTT	CCACCTGAAAATTC
*CFR-1*	GTTCCTCACTATAAGGTGAGTGTAATGA	CTCGTAGACTTTCTATATCAACGATTGGTATT	AACCCAGGAATATCC
*tetJ*	GGGTGCCGCATTAGATTACCTATT	CGTCCAATGTAGAGCATCCATAAT	ATGGCTTGCCCCACCTC

**Figure 1:**
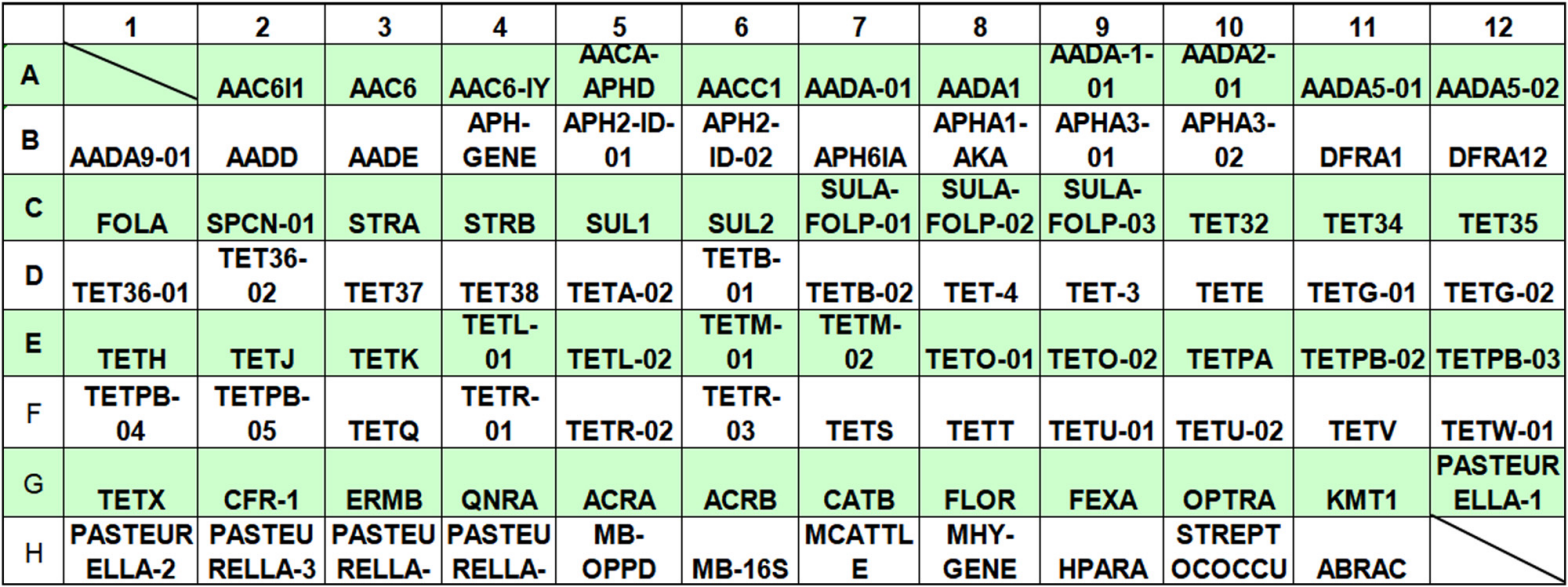
High-throughput detection chip layout board.

After the sequence design of primers and probes was completed, 91 probes and primers were mosaicked into the solid-phase template to construct the high-throughput detection chip designed for this experiment, and the results of the layout plate are shown in Figure 1.

### 3.2 Establishment of TaqMan MGB fluorescence quantitative PCR reaction conditions

**Figure 2:**
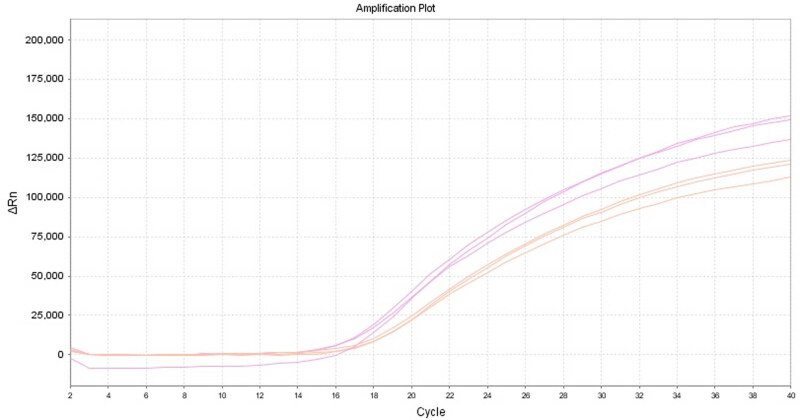
Optimal reaction conditions for TaqMan MGB fluorescence quantitative PCR.

In this experiment, the optimized reaction conditions were established using a panel of 91 constructed plasmid standards as templates. The reaction system was set at 25 μL to obtain a strong signal for selecting the best reaction system and conditions (Table 3, Figure 2). The final selected optimal reaction conditions were as follows: pre-denaturation at 95℃ for 20 s, denaturation at 95℃ for 3 s, annealing and extension at 60℃ for 30 s, with a total of 40 cycles. The optimal reaction system is shown in Tables 1–5.

**Table 3:** Optimum reaction system and conditions

Reagent	Volume (μL)
2 × Taq Man^TM^Fast Advanced Master Mix	12.5
DNA template	1
ddH_2_O	11.5
Total	25

**Table 4:** Repeatability experiment

DNA	Copies		Ct value		Mean Ct	SD	CV (%)
	10^8^	16.78	16.88	16.71	16.76	0.08	0.48
	10^7^	19.62	19.65	19.66	19.64	0.06	0.31
Pm A	10^6^	22.98	22.92	22.94	22.95	0.07	0.34
	10^5^	26.45	26.48	26.44	26.47	0.07	0.24
	10^4^	29.33	29.36	29.38	29.34	0.05	0.17

**Table 5:** Repeatability experiment

DNA	Copies		Ct value		Mean Ct	SD	CV (%)
	10^8^	18.33	18.34	18.36	18.33	0.06	0.46
	10^7^	19.78	19.73	19.77	19.72	0.04	0.37
Mh	10^6^	23.55	23.56	23.58	23.53	0.05	0.32
	10^5^	27.68	27.69	27.63	27.64	0.07	0.51
	10^4^	31.34	31.35	31.37	31.34	0.08	0.43

### 3.3 Drawing standard curves

Using plasmid standards with known copy numbers at concentrations of 1 × 10^10^ copies/μL, 1 × 10^8^ copies/μL, 1 × 10^6^ copies/μL, 1 × 10^4^ copies/μL, and 1 × 10^2^ copies/μL as templates, amplification was performed under the optimized reaction conditions. The standard curves were plotted under conditions where the Ct values were stable.

**Figure 3:**
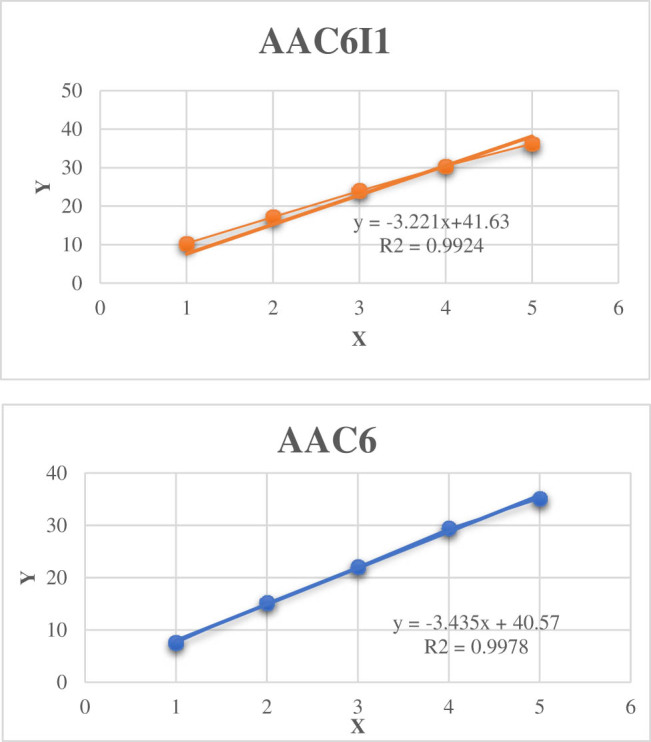
Standard curve of high throughput detection chip AAC6I1, AAC6.

The curve morphology was analyzed, and the correlation coefficients (*R* ^2^) of the standard curves were between 0.99 and 1. The amplification efficiency ranged from 90 to 110%. This indicates that within the concentration range of 10^2^–10^10^ copies/μL, there is a good linear relationship between the logarithm of plasmid concentration and Ct value, and the amplification efficiency is high. Some of the standard curves are shown in Figure 3.

### 3.4 Specific detection

**Figure 4:**
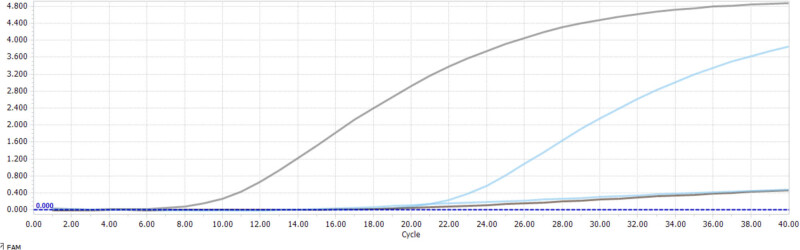
Specific detection of Taq Man MGB fluorescence quantitative PCR.

The results of this experiment showed that only the plasmid DNA of *M. bovis* and Pm in cattle exhibited positive curves, while the remaining pathogenic bacteria showed no amplification curves. This indicates that the high-throughput detection chip that was established has good specificity. Refer to Figure 4 for details.

### 3.5 Sensitivity detection

**Figure 5:**
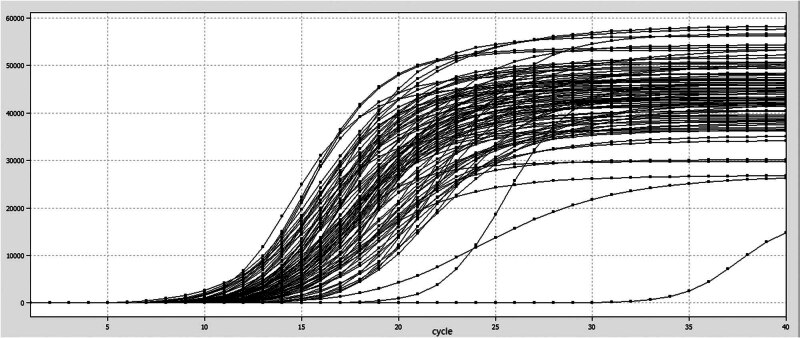
Sensitivity detection of Taq Man MGB fluorescence quantitative PCR (1 × 10^10^ copies/μL).

**Figure 6:**
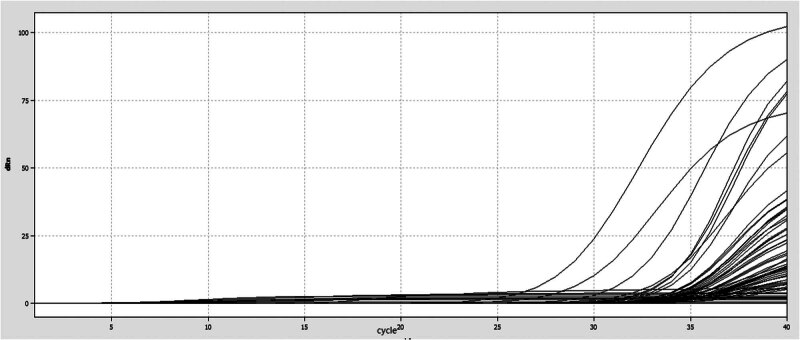
Sensitivity test of Taq Man MGB fluorescence quantitative PCR (1 × 10^1^ copies/μL).

The constructed plasmid standard was diluted in a ten-fold gradient using Elution Buffer. From the detection results, it can be observed that the template with a concentration of 1 × 10^10^ copies/μL showed good amplification curves, while the template with a concentration of 1 × 10^1^ copies/μL exhibited minimal amplification, with Ct values above 40. Therefore, the lower detection limit of the high-throughput detection chip established in this experiment is 1 × 10^1^ copies/μL. Refer to Figures 5 and 6 for details.

### 3.6 Reproducibility testing

Reproducibility experiments were conducted using ten-fold gradient diluted plasmid DNA from bovine Podoviridae Pm and bovine Myoviridae Mh as templates. The experiments were performed three times to evaluate inter-batch and intra-batch reproducibility under the optimized reaction conditions. The results are presented in Tables 4 and 5. According to Tables 4 and 5, the CV% values for the Pm A group ranged from 0.17 to 0.49%, and for the Mh group, the CV% values ranged from 0.31 to 0.5%. All CV% values were below 0.5%, indicating good reproducibility of the constructed high-throughput detection chip.

### 3.7 Clinical sample testing results

**Figure 7:**
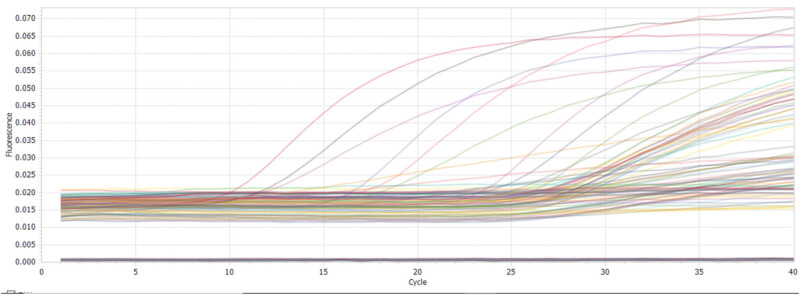
TaqMGB fluorescence quantitative PCR detected 29 clinical positive samples.

Among the 97 throat swab samples collected from the cattle farm, 29 samples were detected as positive, which was consistent with the antimicrobial susceptibility test results from our laboratory. Among these positive samples, 11 samples showed resistance to aminoglycoside drugs, 9 samples showed resistance to sulfonamide drugs, 5 samples showed resistance to chloramphenicol drugs, and 4 samples showed resistance to tetracycline drugs. Additionally, two pathogenic bacteria, bovine Podoviridae Pm and bovine *M. bovis*, were detected. Refer to Figure 7 for more details.

## 4 Discussions

BRD has been a persistent and pervasive challenge in the cattle industry, with its tentacles reaching both local and global scales, substantiated by a myriad of studies and surveys. A striking revelation from a survey conducted by Zhao et al. [22] underscores the severity of the situation in China, where the infection rate in beef cattle farms soars to over 90%, and the mortality rate is staggeringly high at 35%. This not only poses a substantial threat to the livelihoods of farmers but also casts a shadow over the sustainable development of the entire cattle industry. In the context of China, BRD predominantly manifests as a mixed infection, notably involving bovine *M. bovis* and bovine capsular type A Pm, presenting a unique challenge in understanding and managing the disease in this region.

Internationally, the scenario is equally concerning. Research from Belgium and the United Kingdom [23–25] has not only highlighted the prevalence of BRD but also brought to light the isolation rates and incidence percentages, which are indicative of the disease’s significant impact in these areas. The current methodologies for diagnosing BRD and testing for drug resistance predominantly hinge on conventional methods. These traditional approaches, while reliable, are inherently time-consuming and labor-intensive, thereby inhibiting their efficacy in providing swift and effective clinical interventions.

In this study, a novel approach was adopted, developing a TaqMan MGB fluorescence quantitative detection method. This method, which involved the crafting of specific probes and primers for the primary pathogen and 81 related drug resistance genes of BRD, demonstrated a promising sensitivity of 1 × 10^1^ copies/μL and a SD of less than 0.5%. This method not only showcased superior specificity and repeatability but also outperformed the multiplex PCR method for detecting BRD, established by Jeon et al. [26], in terms of sensitivity. When applied to clinical samples, this method identified a positivity rate of 30% from 97 nasal swab samples, surpassing conventional detection methods in both speed and efficacy by providing results within 1 h. This marks a substantial advancement compared to previous gene chip technology [27] and other methods like the multiplex PCR method [28–30], which necessitated considerably longer durations to yield results.

Simultaneously, this study engineered and synthesized nine specific primers and MGB probes, targeting bovine capsule type A Pm and bovine *M. bovis* QRDR mutation sites. The quantitative analysis of the chip revealed that both the standard curve and amplification efficiency met satisfactory levels. The method’s specificity was rigorously tested, demonstrating robust specificity as only the positive samples produced an amplification curve. Further verification confirmed the minimum detection limit of plasmid DNA to be 1 × 10^4^ copies/μL. The method exhibited stability, evidenced by the in-batch and inter-batch CV values being below 2%. In clinical sample detection experiments, the chip showcased its advantageous attributes, enabling the rapid, efficient, and accurate detection of QRDR target mutations, with its genotype aligning consistently with the drug resistance phenotype.

## 5 Conclusions

In the pursuit of advancing the management and control of BRD, this study introduced a pioneering detection chip, leveraging TaqMan MGB probes and focusing on the sequences of major BRD pathogens and drug resistance genes. While the chip exhibits promising capabilities, particularly in conducting drug resistance profiling of clinical strains and potential future applications in pathogen identification, it is imperative to acknowledge its limitations and envision future research trajectories.

A palpable limitation of the current study revolves around the production cost of the developed chip, which stands as a substantial barrier to its widespread clinical application. This economic constraint necessitates a meticulous exploration into alternative manufacturing processes and materials that could potentially mitigate costs without sacrificing the chip’s efficacy and reliability. Moreover, while the chip is grounded in the sequences of predominant BRD pathogens and drug resistance genes, it is crucial to consider the dynamic nature of microbial genomes and resistance mechanisms, which may necessitate ongoing updates and validations to ensure its sustained relevance and accuracy in detection.

Peering into the future, the chip could indeed serve as a foundational tool in fortifying the management of BRD across cattle farms, transcending its current applications and morphing into a pivotal asset in BRD prevention and control. Future research could delve into enhancing the chip’s accessibility and applicability, ensuring it can be seamlessly integrated into various operational scales and contexts within the cattle farming industry. Furthermore, forging collaborations with diverse stakeholders, spanning cattle farmers, veterinary professionals, and policymakers, could unearth invaluable insights into the chip’s practical applications and potential challenges in broader implementation. Such collaborations could also facilitate the co-creation of strategies to navigate these challenges, ensuring that the chip is not only a scientific innovation but also a practical, real-world solution to managing BRD.

In essence, while the developed chip marks a significant stride toward more efficient and precise management of BRD, it is the amalgamation of ongoing scientific innovation, practical adaptability, and collaborative efforts that will truly propel its impact in the field, fostering a future where BRD can be effectively diagnosed, managed, and potentially mitigated across the global cattle farming landscape.
